# Novel nondelay-based reservoir computing with a single micromechanical nonlinear resonator for high-efficiency information processing

**DOI:** 10.1038/s41378-021-00313-7

**Published:** 2021-10-20

**Authors:** Jie Sun, Wuhao Yang, Tianyi Zheng, Xingyin Xiong, Yunfei Liu, Zheng Wang, Zhitian Li, Xudong Zou

**Affiliations:** 1 The State Key Laboratory of Transducer Technology, Aerospace Information Research Institute, Chinese Academy of Sciences, Beijing, China; 2 School of Electronic, Electrical and Communication Engineering, University of Chinese Academy of Sciences, Beijing, China; 3 QILU Aerospace Information Research Institute, Jinan, China

**Keywords:** Sensors, Electronic devices

## Abstract

Reservoir computing is a potential neuromorphic paradigm for promoting future disruptive applications in the era of the Internet of Things, owing to its well-known low training cost and compatibility with hardware. It has been successfully implemented by injecting an input signal into a spatially extended reservoir of nonlinear nodes or a temporally extended reservoir of a delayed feedback system to perform temporal information processing. Here we propose a novel nondelay-based reservoir computer using only a single micromechanical resonator with hybrid nonlinear dynamics that removes the usually required delayed feedback loop. The hybrid nonlinear dynamics of the resonator comprise a transient nonlinear response, and a Duffing nonlinear response is first used for reservoir computing. Due to the richness of this nonlinearity, the usually required delayed feedback loop can be omitted. To further simplify and improve the efficiency of reservoir computing, a self-masking process is utilized in our novel reservoir computer. Specifically, we numerically and experimentally demonstrate its excellent performance, and our system achieves a high recognition accuracy of 93% on a handwritten digit recognition benchmark and a normalized mean square error of 0.051 in a nonlinear autoregressive moving average task, which reveals its memory capacity. Furthermore, it also achieves 97.17 ± 1% accuracy on an actual human motion gesture classification task constructed from a six-axis IMU sensor. These remarkable results verify the feasibility of our system and open up a new pathway for the hardware implementation of reservoir computing.

## Introduction

Recently, emerging sensor applications, such as the Internet of Things (IoT)^1^ and ubiquitous sensing, require sensors with smaller size and lower power consumption, as well as “edge computing”^2^ capabilities, to process a deluge of data locally. These expanding computing requirements have motivated the creation of new and specialized computing paradigms to break through the “von Neumann bottleneck”. Among them, neuromorphic computing that mimics a biological neural network has been advocated as a candidate in recent years because of its high energy efficiency^3^. As a neuromorphic computing paradigm, reservoir computing (RC)^4–6^ was originally a recurrent neural network (RNN)^7–9^ framework and is therefore suitable for temporal information processing. RC is different from conventional RNNs in that the weights on the recurrent connections in the reservoir are not trained; only the output connection weights in the readout are trained, which makes it possible to drastically reduce the computational cost of learning. More importantly, the hardware implementation of RC can be achieved using a variety of nonlinear dynamic systems with nonlinearity and fading memory (or short-term memory). Because a mechanism for adaptive changes is not necessary for training, the main two implementation structures are RC based on numerous randomly interacting nonlinear nodes and a time-delayed nonlinear system^10^.

RC based on spatially extended nodes provides efficient parallel information processing^11,12^. However, it suffers from the complexity of hardware implementation. Simple RC based on a time-delayed nonlinear system possessing only a single nonlinear node has been proposed. It can emulate the spatially extended nodes of RC using virtual nodes temporally extended along with a delayed feedback. This superiority has recently motivated the search for hardware implementation using emerging devices, such as electronic devices^13^, optical systems^14–19^, spintronic devices^20^, dynamic memristors^21–25^, and mechanical resonators^26,27^. However, the delayed feedback and the additional masking procedure reduce processing efficiency. Recent studies have aimed to solve this problem by optimizing the system parameters^13,28^, such as mask length and feedback strength, or using different feedback structures, such as double feedback loops^16^ and parallel multiple feedback loops^29,30^. However, the effect of the nonlinear characteristics of the device on the performance of the RC system has rarely been studied. Moreover, an initial report demonstrated the feasibility of RC with a single “delay-coupled” nonlinear microelectromechanical system (MEMS) resonator, and its best classification accuracy was only 78+2% for the TI-46 recognition benchmark.

In this work, we propose a novel reservoir computer structure using a single micromechanical resonator with hybrid nonlinear dynamics and omitting time-delayed feedback. Moreover, we focus on the well-known nonlinear dynamics of the micromechanical resonator and first propose a hybrid nonlinear response (HNL), which comprises the transient nonlinear response (TNL) and the Duffing nonlinear response (DuNL). Due to the dynamic richness of the HNL, time-delayed feedback can be removed to achieve high-efficiency RC. Furthermore, we define a self-masking process to replace the traditional masking procedure to simplify and improve the efficiency of RC. The self-masking process directly feeds serialized input data into the reservoir, reshaped by the e-exponential characteristics of TNL with a certain temporal solution, and then picks up the nonlinear cumulative response at the separation time. Since our mask procedure utilizes the self-nonlinear characteristics of the reservoir, the masking procedure and RC are simultaneously completed, which is why we call it a self-masking process.

This allows us to achieve a novel RC architecture using the HNL with the self-masking process for high-efficiency temporal pattern classification, such as the Mixed National Institute of Standards and Technology (MNIST) handwritten digit task, TI-46 spoken word recognition benchmark, and human motion gesture recognition task sensing, from a six-axis inertial measurement unit (IMU) sensor. To evaluate the memory capacity of our system, we perform a nonlinear autoregressive moving average (NARMA) task. The results show that this novel structure can effectively adjust the nonlinear richness of the system to adapt to the specific pattern classification task^13^; it also reduces system multiparameter optimization difficulties and simplifies control loop complexities. More importantly, the simple structure and device compatibility with MEMS can facilitate the hardware implementation of RC and promote the emergence of disruptive applications using MEMS technology in the future IoT era.

## Results

### Hybrid nonlinear resonator-based RC system

**Fig. 1:**
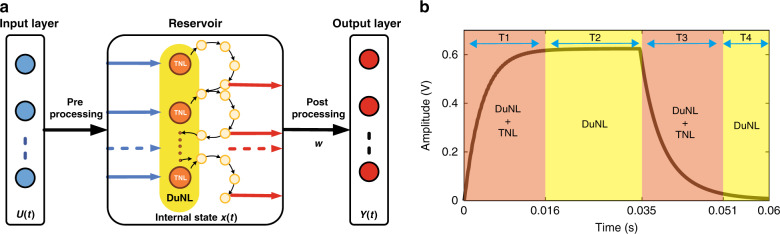
RC system architecture and hybrid nonlinear phenomena. **a** Scheme of the hybrid nonlinear resonator-based RC system. The duration $t = \tau = \theta \cdot N$ is the total length of the input sequences, *θ* is the separation time between input signal data, and *N* is the number of neural nodes. ${{{\boldsymbol{x}}}}\left( t \right) = f({{{\mathbf{U}}}}({{{\mathrm{t}}}}))$, *f* is the hybrid nonlinear function constructed with TNL and DuNL, and the output nodes are linear weighted sums of the reservoir states, which are given by the value $y\left( t \right) = {{{\mathbf{w}}}}^{{{\boldsymbol{T}}}}{{{\boldsymbol{x}}}}\left( t \right)$, where **w** is a vector of weights and *y*(*t*) is the target value. The dark brown circles with the label “TNL” represent the neural nodes of the reservoir, and the light yellow circles represent a series of interconnection “virtual nodes” contained in a neural node. **b** Hybrid nonlinear time-domain response of the resonator. Here the input sequence is a periodic sine wave ($V_{{\rm{ac}}} = 1\,{{{\mathrm{V}}}}$, $f_{\rm{d}} = 184.2\,{{{\mathrm{KHz}}}}$, *t* = 0.035 s), and the responding current is recorded by the data acquisition and processing circuit.

In the time-delayed RC, structural parameters such as the mask function, the number of virtual nodes, and feedback strength should be optimized to generate a sufficiently rich reservoir state, which reduces processing efficiency. In particular, to obtain a large number of different transient responses to the input, the input signal is time-multiplexed by a mask function that serves the dual purpose of serializing the input and maximizing the effectively used dimensionality of the system. Therefore, we propose hybrid nonlinear RC with a self-masking process. The basic principle of our scheme is shown in Fig. 1a. It is composed of three distinct parts: an input layer, a reservoir, and an output layer. The serialized input signals are fed to the reservoir after preprocessing. Then the self-masking process and nonlinear transformation are simultaneously realized in the hybrid nonlinear reservoir. Thus, the reservoir states are sampled through postprocessing, and the training and test procedures are implemented using a linear regression algorithm. Compared with time-delayed feedback RC^13^, we directly serialize the input stream *U*(*t*) and feed it into the reservoir, and different degrees of nonlinear cumulative effects can be obtained by the self-masking process, which simplifies the masking procedures and improves the information processing efficiency.

The concept of HNL we introduce ensures the rich nonlinear dynamics of the reservoir. Figure 1b shows the envelope detection result of the HNL oscillation response of the resonator, which can be roughly divided into four stages: T1 represents the oscillation starting stage, T2 the steady-state oscillation stage, T3 the oscillation attenuation stage, and T4 the oscillation resting stage. For T1–T4, all operate in the Duffing nonlinear oscillation state of the resonator, but the T1 and T3 stages also operate in the TNL. We select *θ* < *T* (*T* = T1 = T3) for better state richness due to the HNL we propose. Thus, the masking procedure in time-delayed RC can be replaced with the self-masking process, and the feedback loop is not necessary. As a result of this simple structure, the system is capable of generating a sufficiently rich reservoir state for high-efficiency information processing.

### Model analysis of hybrid nonlinear RC

**Fig. 2:**
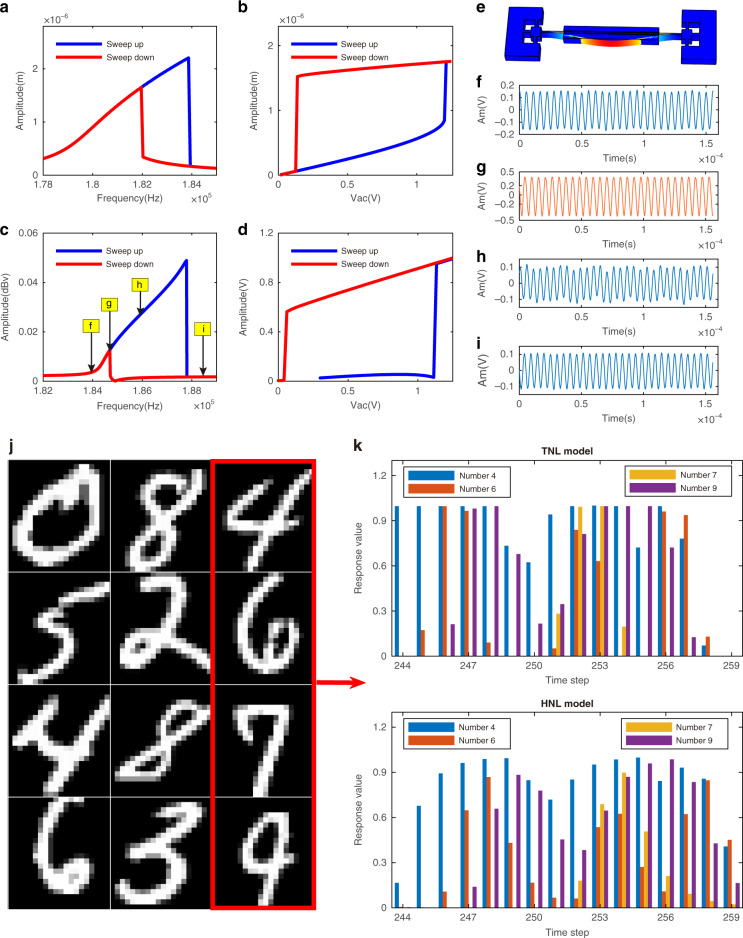
Nonlinear resonator and RC models. **a**, **b** Simulation results of the frequency sweep and amplitude sweep of the resonator. Left: response of the resonator to frequency sweeping for *Q* = 4300, polarization voltage *V*_dc_ = 30 V, and excitation amplitude *V*_ac_ = 1 V; right: amplitude sweeping for a fixed drive frequency of *f*_d_ = 182 KHz. **c**, **d** Open-loop experiment test results of the frequency sweep and amplitude sweep of the resonator under the same conditions. The optimal driving frequency is *f*_d_ = 184.2 KHz at the *g* point, and the resonator natural frequency is *f*_n_ = 175 KHz. **e** FEA model of the beam showing its first mode shape. **f**–**i** Time domain response at different frequencies. **j** Some examples from the MNIST database. **k** Classification results of two RC models. The reservoir states are significantly different after the two different models corresponding to the number “4-6-7-9.”

**Fig. 3:**
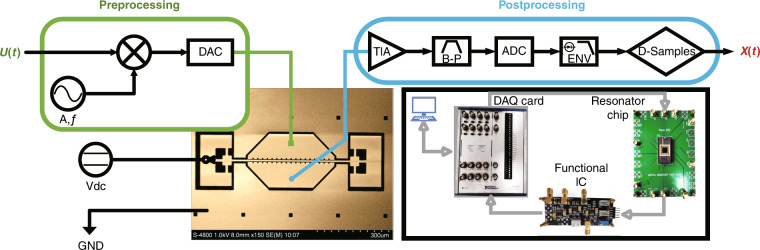
Schematic of the experimental reservoir computer. The experimental set-up of the control system and the scanning electron microscope (SEM) image of the resonator. The doubly clamped silicon beam resonator can be actuated by the driving electrode (the green solid block) and read by the sensing electrode (the blue solid circle) simultaneously; polarization voltage *V*_dc_ is added to the beam (the black hollow circle with arrow) so that the parallel plates form a stable potential.

In this hybrid nonlinear RC system, a microelectromechanical clamped–clamped (C-C) silicon beam resonator is used as the reservoir to nonlinearly map the input data into a higher-dimensional state space, which can also be seen as a typical underdamped second-order oscillation system. This hybrid nonlinear reservoir combines the DuNL characteristics of the resonator^31,32^ and its transient exponential nonlinear response characteristics as a typical second-order oscillation system, which guarantees the rich nonlinear dynamics of single resonator RC to process the pattern classification tasks. The resonator is driven and detected by the parallel plate electrostatic force. A scanning electron microscopic (SEM) image of the resonator is shown in Fig. 3, and its displacement can be approximated by the Duffing nonlinear equation:

1 $$\ddot x + 2\xi w_n\dot x + w_n^2x + \beta x^3 = F_{\rm{d}}(t),$$

where *x*, $\dot x$, and $\ddot x$ are the displacement, velocity, and acceleration of the resonator, respectively, $w_{\rm{n}} = 2\pi f_{\rm{n}}$ is the natural angular frequency of the resonator in its linear regime, $\xi = \frac{1}{{2Q}}$ is the damping ratio, *Q* is the quality factor, and *F*_d_ is the force per unit mass driving the beam. Note that *β* is the coefficient controlling the amount of nonlinearity in the restoring force and introduces the Duffing nonlinearity to the equation. In the case of the C-C beam (Figs. 2e and 3), the value of *β* can be estimated by^32^

2 $$\beta = \frac{{32E}}{{\surd 2\rho L^4{{{\mathrm{A}}}}}}$$

where *E* is the silicon Young’s modulus, *L* is the beam length, *ρ* is its density, and *A* is a constant term. Equation (2) further indicates that the geometric nature of the nonlinearity of the resonator depends on the beam length. Short beams can cause a larger nonlinear restoring force term than long beams but need a larger excitation amplitude.

The size information of the (C-C) silicon beam resonator is described in the “Device fabrication” part, which is designed to satisfy the demands for the appropriate value of *β* and *Q*. To drive the C-C beam to the sufficient nonlinear region with less energy consumption, an expected *β* value of 2.4 × 10^22^ Hz^2^ m^−2^ with an effective beam length of 500 µm should be determined. If *β* = 0, the beam will oscillate periodically in a linear region, and we can obtain the analytical solution $x\left( t \right) = 1 \pm \frac{{e^{ - \frac{{w_{\rm{n}}}}{{2Q}}t}}}{{\sqrt {1 - \left( {\frac{1}{{2Q}}} \right)^2} }}$. When assuming the initial condition *F*_d_ = 0, this e-exponential term is introduced as the TNL. The HNL provides sufficiently rich computing dynamics when mixing the *β* and e-exponential terms. In addition, the natural frequency and quality factor also influence the performance of RC. To maintain a certain memory capacity, a high quality factor *Q* >1000 should be considered, which determines the decay time for the TNL. To further reduce the “anchor loss”, a unique “cross” anchor design is adopted.

In the hardware implementation of time-delayed RC by a mechanical oscillator^26,27^, a high natural frequency and relatively low value quality factor must be combined for a higher processing speed ${{t}}_0^{ - 1} = \frac{{\pi f_{\rm{n}}}}{{MQ}}$, where *M* is the number of virtual nodes. Generally, several hundred virtual nodes^28^ (*M* ~ 400) are employed to obtain good performance; thus, the system needs a larger driving voltage to operate in suitable nonlinear states because of the low quality factor (*Q* ~ 100). While the number of virtual nodes *M* is not needed in the self-masking process, a high quality factor (*Q* ~ 4300) can be chosen, which not only ensures the processing speed but also improves the nonlinear effect of the TNL.

As mentioned above, the characterization of the nonlinear dynamic response of the beam is crucial since it is the source of reservoir nonlinearity. A “frequency sweep” and “amplitude sweep” are common characterization methods used to analyze the nonlinear response of a beam; therefore, we construct a numerical simulation of Eq. (1) to determine several main parameters, which are used to drive the resonator into an appropriate nonlinear state for the realization of reservoir state richness, such as the driving frequency *f*_d_ and the excitation amplitude.

The Duffing nonlinear frequency/amplitude response can be observed in Fig. 2a, b, and the nonlinear solutions to (1) equation of motion have been well studied previously^32^. Finite element analysis is performed through the solid mechanics interface of COMSOL Multiphysics 5.3a to further simulate the dynamic vibration modes of the beam. Figure 2e shows the first mode shape. Moreover, to ensure that the beam works in a higher signal-to-noise ratio and stability amplitude output at the specified drive frequency, the value at the front bifurcation point (g point) of the frequency hysteresis loop is selected^33^, as shown in Fig. 2c. The sweep results of the simulation are almost the same as the experimental results (Fig. 2c, d), verifying the feasibility of the constructed model. Therefore, we can choose suitable parameters and states to verify our HNL-RC concept.

**Fig. 4:**
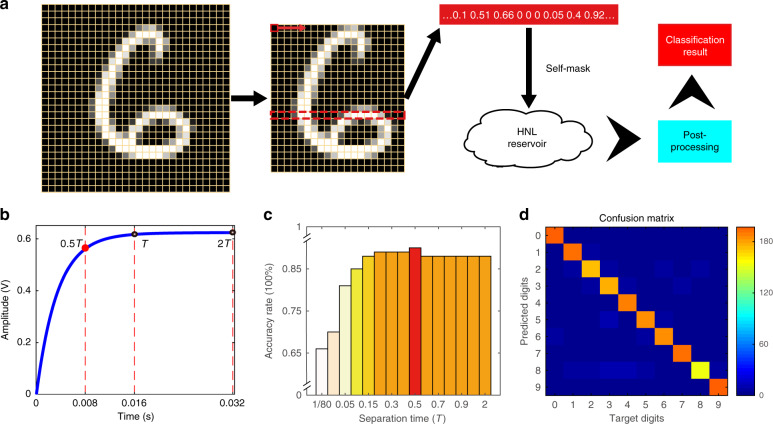
Handwritten digit recognition using an HNL resonator-based RC system. **a** Schematic diagram of handwritten digit classification. **b** HNL time-domain response curve of the resonator. The decay time *T* is equal to T1 or T3 in this curve, and the black circle indicates different separation times, *θ*. **c** Optimal parameter *θ* experiment results. The plot of the MNIST subdataset classification accuracy versus the separation time *θ* in Fig. 4b. The test conditions of the experiment are $f_{\rm{d}} = 184.2\,{{{\mathrm{KHz}}}},\;V_{{\rm{dc}}} = 30\,{{{\mathrm{V}}}},\;V_{{\rm{ac}}} = 1\,{{{\mathrm{V}}}},\;T = 0.016\,{{{\mathrm{s}}}}$. **d** False color confusion matrix showing the predicted results from the resonator-based RC system versus the target outputs. The color bar on the right side of the figure is the color distribution value after the normalization operation, and the color blocks in the matrix correspond to it. The classification accuracy is 93%, and the specifics of the training procedure are detailed in the “Methods” section.

After determining the key parameters of the beam, we choose the handwritten digit recognition dataset to compare the classification performance of the TNL- and HNL-RC models. We choose a subdataset to test or optimize the system, which contains 1000 samples with 10 classes randomly selected from the MNIST dataset^34^: 100 samples for the test set, and 900 samples for the training set. Preprocessing is performed before the samples are input to the reservoir to reduce redundant information of the input signal, as shown in Fig. 4a. The details are shown in the “Methods” section.

Figure 2k shows the reservoir states corresponding to the four test samples shown in Fig. 2j for the two different models. The reservoir states of the two models are significantly different, preliminarily verifying the rationality of the above analysis. The reservoir state is then used as input to the readout function via ridge regression for training and classification. The better temporal information processing ability of the HNL reservoir is clearly revealed by recognizing the test dataset; the classification accuracy rate of the TNL model is 88% and that of the HNL model is 91%. We thus demonstrate that the novel RC structure possesses highly efficient information processing capabilities and good classification accuracy.

### Experimental set-up for single-resonator RC

When verifying the concept of the hybrid nonlinear RC structure by the simulation, hardware implementation experiments should be further established to verify the feasibility of the system. We select the widely studied MEMS C-C beam resonator, and its nonlinear oscillation characteristic is verified through numerical simulation and yields similar results. The SEM image and the experimental set-up are shown in Fig. 3.

In the experimental set-up of our system, the hardware implementation of the time-delayed RC^26^ is simplified because of the self-masking process, including the multiplier applied to multiply input digital data *U*(*t*) with a mask. The adder is used to add the feedback signal, the shift register, and amplifier for the precise time delay, which are no longer required in our system. When the sensing electrode obtains the state response, an envelope filter (ENV) and a downsampling module (D-Samples) are set behind the analog-to-digital converter (ADC) because it is convenient to adjust the response waveform under different nonlinear response conditions using the LabVIEW program. Therefore, the experimental set-up of the single resonator RC better simplifies the complexity compared with the time-delayed feedback RC and improves the flexibility and information processing efficiency.

### MNIST handwritten digit recognition

The nonlinear cumulative effect during the self-masking process can be adjusted by varying the parameter *θ*, ${\theta} = n \cdot {{T}}\left( {n \in \left[ {0,1} \right]} \right)$, where ${{T}} = \frac{{2Q}}{{w_{\rm{n}}}}$ is the decay time of the resonator. Here we ignore the influence of the decay time itself because *T* is not changed under the specific experimental conditions. According to previous research^13,26^ and our simulation analysis, the separation time value is set at $\theta \sim \frac{1}{2}T = 0.008\,{\rm{s}}$ to offer optimal performance. A parameter optimization trial is designed by changing the parameter *θ* with the subdataset, and the results are shown in Fig. 4b, c. We can obtain a classification accuracy of 67% when $\theta = \frac{1}{{80}}{{T}}$; increasing the separation time to $\frac{1}{2}{{T}}$ can potentially achieve 91% accuracy, and the accuracy can be lowered to 88% as *θ* continues to increase because the steady-state oscillation stages only have one nonlinear response. Consequently, we experimentally verify that the richness of the nonlinear dynamics of the reservoir can be effectively optimized by varying the separation time *θ* so that it can be more widely and effectively applied to different types of classification tasks with this hybrid nonlinear RC system.

In addition, after parameter optimization and selection of specific nonlinear vibration states, it is vital to further verify that the system is suitable for MNIST tasks with a large amount of data. We randomly select 25,000 samples from the MNIST dataset, of which 2500 samples are used as the test set and 22,500 samples are used as the training set. When we set the separation time $\theta = \frac{1}{2}T$, the final classification accuracy obtained from the RC system is 93%. Figure 4d shows a false-color confusion matrix highlighting the experimentally obtained classification results from the RC system versus the desired outputs.

### NARMA task to assess memory capacity

Different tasks require different key properties to make a correct estimation of the target function. While classification tasks require a strong nonlinear transformation^13^, forecasting tasks are strongly dependent on good linear memory. In the experiments assessing the handwritten digit recognition task, we demonstrate the high-efficiency classification performance of the HNL reservoir. Here we can also verify the memory capacity of this reservoir through the NARMA benchmark.

NARMA is an acronym for the nonlinear autoregressive moving average. It is one of the most widely used benchmarks for measuring memory ability. The parameter *n* represents the correlation between the current and the previous *n* data. We choose the task parameter *n* = 1, as the nearest-neighbor correlation in this reservoir framework only exists between the virtual nodes (the details are presented in “Methods”). Based on the research results^13^, we should choose a weak nonlinearity condition to obtain better forecasting precision by selecting a suitable *θ*, which is not smaller than *T*. Another option is reducing the parameter *Q* to weaken the Duffing nonlinear effect. Formula (3) is the transfer function of the NARMA1 task, described as follows:

3 $$y_k = 0.3y_{k - 1} + 0.05y_{k - 1}^2 + 1.5u_k^2 + 0.1.$$

where *k* is the length of the training and test sequences, and the input *u*(*k*) is generated from a uniform density in [0,0.5].

**Fig. 5:**
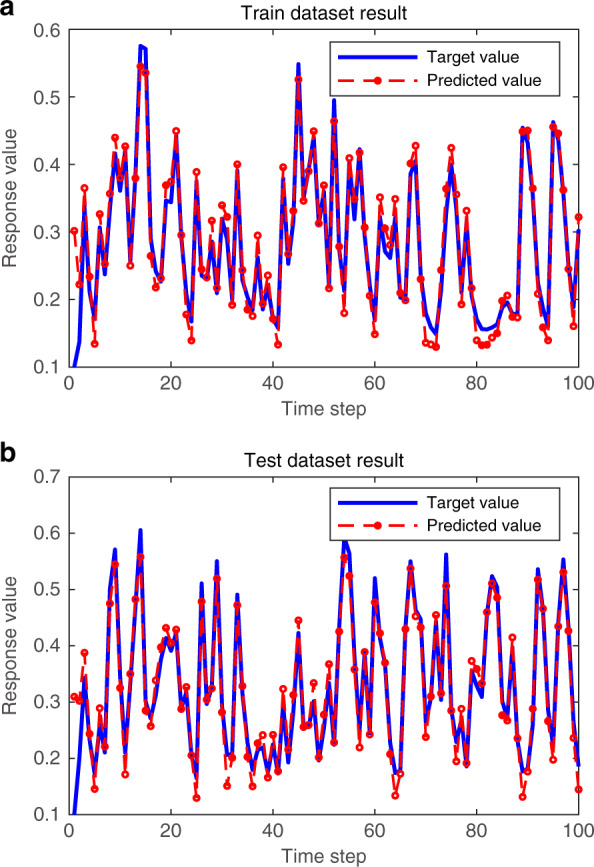
NARMA prediction task. **a** is the training set results, and **b** is the test set results. When we set *k* = 300, the training/test input contains 300 random data sequences. The figure shows the results of 100 intercepted data points, ~*T*, and the other parameters are the same as above.

To quantify the performance of the reservoir, the normalized mean square error (NMSE) of the predicted value versus the value obtained from the NARMA model is used. The details are described in the “Methods” section. In Fig. 5, we depict the predicted results versus the target value. The training set result is NMSE = 5.5e-3, and the test set result is NMSE = 0.051. Therefore, we achieve memory capacity in the HNL reservoir but have the potential to realize a longer memory capacity with the novel architecture.

### Motion gesture recognition of six-axis IMU sensor

To further verify the high-efficiency information processing ability of the proposed HNL-RC system for the real sensing of temporal signals and demonstrate its application potential in real-world scenarios, we design an application scenario to recognize the different human motion gestures by our proposed HNL-RC system. Signal data acquisition from a homemade six-axis IMU sensor, which integrates commercial three-axis accelerometers and three-axis gyroscopes, is performed using the functional integrated circuits made by our research group.

**Fig. 6:**
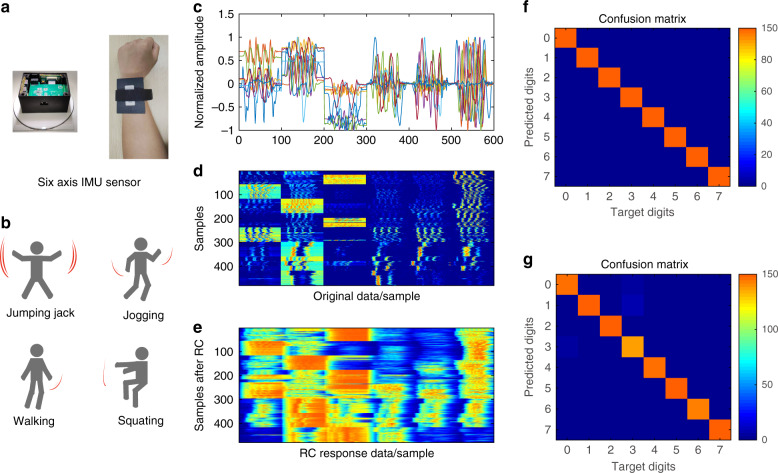
Motion gesture recognition of the six-axis IMU sensor using the HNL-RC system. **a S**ix-axis IMU sensor and wearing. **b** Schematic diagram of different motion gestures. **c** Normalized time-domain signal data after acquisition and preprocessing. **d**, **e** The color block diagram shows the entire sample-set original data and the response results after the HNL-RC system. **f**, **g** False-color confusion matrix showing the predicted results versus the target outputs, **f** shows the result of the sample set with only one certain conner, and **g** contains three different subjects.

Figure 6a, b shows the optical image of the six-axis IMU sensor and wearing effect and the samples of four out of eight different motion gestures, which include jumping jacks, jogging, walking, squatting, stretching, chest expansion, arm circling, and body circling. Figure 6c shows the response of the sensor when the subject performs the eight gestures. The preprocessing involves only smooth filtering with a 30-point window length to reduce the noise, sampling, and normalization of each waveform. The final signal contains 600 feature points. We train and test the system with the motion gesture sample set, which consists of 8 actions, each repeated 20 times, for a total of 3 subjects. After obtaining the response of the HNL-RC, as shown in Fig. 6d, e, tenfold cross-validation is used to obtain the optimal weight matrix to prevent the system from overfitting to specific training and test data. The experimental conditions are the same as above, and the input voltage streams with 10 different time intervals *θ* (0.05*T*, 0.1*T*, 0.2*T*, 0.3*T*, 0.4*T*, 0.5*T*, 0.7*T*, 0.9*T*, *T*, 2*T*) and the optimal *θ* = 0.2*T*.

Figure 6g shows the best recognition results obtained by the HNL-RC system; it can achieve (97.17 ± 1)% recognition accuracy for the real sensing signal from the six-axis IMU sensor. Moreover, if the application scenario is changed to only the motion gesture recognition of a certain person, a classification accuracy rate of (99.29 ± 0.5)% can be obtained by using this system. Therefore, the single resonator-based HNL-RC system is verified as a new architecture with high efficiency information processing ability.

## Discussion

The remarkable performance demonstrates the high-efficiency information processing ability for pattern recognition tasks and short-term memory capacity for simple forecasting tasks, which shows the feasibility of our system and opens up a new pathway for the hardware implementation of RC. The use of a hybrid nonlinear system can simplify the hardware reservoir implementation of RC, and it can improve the computation rate compared with traditional time-delayed architectures.

To further illustrate the excellent performance and reliability of the new architecture proposed here, we test the MNIST handwritten digit recognition benchmark by our HNL-RC system based on a single resonator, and the classification accuracy is better than that of memristor-based RC, which uses 88 memristors^24^. Furthermore, a “similar” preprocessing procedure is performed in the TI-46 spoken word classification task. We construct a time-delayed feedback reservoir system using the same resonator to compare the HNL-RC system, and the experimental results show that the classification accuracy of the latter (87.4%) is superior to that of the former (78%). All of these results verify the high efficiency and accuracy pattern recognition ability of this novel HNL-RC architecture. The NARMA benchmark verifies the regression forecasting ability when *n* = 1 for the memory capacity, which we will improve in future work. Then we design a motion gesture recognition experiment to test the feasibility of the architecture. The sample set is composed of a real signal sensed from a six-axis IMU sensor or three accelerometers. The high classification accuracy in the “Results” section proves the high-efficiency signal classification capability, and it also provides basic performance verification for future “sensing + computing” integrated device applications in this novel hybrid nonlinear RC hardware system.

## Conclusion

In summary, we propose a novel RC architecture using a single micromechanical resonator with hybrid nonlinear dynamics while omitting time-delayed feedback. Based on this approach, we numerically and experimentally analyze the nonlinear response of the resonator and first propose that the hybrid nonlinear dynamics of the resonator comprise hybrid types of nonlinear responses, transient responses, and Duffing responses. Moreover, a self-masking process is defined based on the approach. We also perform two typical tasks and one real signal sample-set task sensed by a self-assembled six-axis IMU sensor to verify its classification capability and memory capacity. Experimental tests on the MNIST dataset show a high accuracy of 93% for handwritten digit classification, the motion gesture classification accuracy of the sample set composed of three subjects is 98.17%, and the accuracy can reach 99. 79% when the sample set is composed of only one subject. For the NARMA task, the results show that the NMSE is 0.051 when the correlation parameter *n* equals 1, which is consistent with the situation wherein only the nearest-neighbor input is correlated in the reservoir.

Considering the simple structure of our system and the device compatibility with MEMS, we expect that the proposed novel structure can facilitate the hardware implementation of RC and inspire emerging applications using MEMS technology in the future IoT era.

## Methods

### Device fabrication

The C-C beam resonator is microfabricated on (100) p-doped silicon on a glass substrate by the standard silicon-on-glass process. A device layer thickness of 40 µm defines the width of the beam; the length, in-plane thickness, and the gap between the beam and the drive/sense electrode are chosen to be 500, 6.5, and 3 µm, respectively, and the electrode length is 360 µm. For more complex nonlinearity of the resonator, we select the parallel plate drive and detection mode instead of the comb drive mode. For the COMSOL simulation diagram and the actual device diagram of the designed resonator, please refer to Figs. 2e and 3, respectively.

### Device characterizations

The experimental single resonator RC system is realized with a personal computer (PC), an NI 6366 X Series Data Acquisition (SDA), and a resonator device with a functional interface circuit (IC). The PC is used to run the loop of the control algorithm, which is programmed by LabVIEW 17.0; the SDA is used to realize the function of an ADC and a digital-to-analog converter, which are 12 bits, and the functional IC contains a trans-impedance amplifier module, a second amplifier module, and a bandpass filter module for transforming, amplifying, and filtering the response signal, respectively.

Suitable driving parameters should be chosen before the final test with special tasks. A Zurich lock-phase amplifier is the most commonly used basic performance measurement instrument for MEMS devices. It is used to perform the open-loop frequency scanning test to determine the required driving frequency and the effective quality factor.

### Mixed National Institute of Standards and Technology

The MNIST database^34^ is a large dataset that is commonly used for training and testing classification capacity. The database was created by “remixing” the digit samples written by high school students and employees of the United States Census Bureau and consists of 60,000 training samples and 10,000 test samples. Each sample in the dataset is composed of a 28 × 28 gray value matrix. Preprocessing was performed before the images were fed into the reservoir, as shown in Fig. 4a. Taking the image of 6 as an example, the original grayscale image of 28 × 28 pixels was trimmed to a 22 × 20 pixel image to reduce redundant information. Then the 22 × 20 pixel matrix was transformed into 1 × 440 temporal sequences of input pulse streams with separation time *θ*, serializing the *N* = 440 input signal as the “neural” nodes of the HNL reservoir.

Finally, we obtained a 440 × 10 readout network that was used for classification after training. To perform the MNIST classification function, we need to construct ten appropriate target functions as ten linear classifiers, each of which is a polynomial function composed of the optimal weight coefficient vector, ${{{\mathrm{y}}}}_i({{{\mathrm{t}}}}) = {{{\boldsymbol{w}}}}_i^T{{{\boldsymbol{x}}}}(t)$ with *i* = 10. For every test sample, the function is applied to select the actual digit through a winner-takes-all approach. The target function is +0 if the handwritten digit does not correspond to the sought digit and +1 if it does. We called this postprocessing.

### NARMA task

The NARMA task is one of the most widely used benchmarks for measuring RC memory capacity. The full name is the nonlinear autoregressive moving average^13^. It is used in many other publications in the context of RC, such as refs. ^5,35^. For the NARMA task, the input *u*(*k*) is generated from a uniform density in [0,0.5]. Then, after being normalized, the variable *n* is a positive integer value of [1,∞], where a larger *n* represents a stronger correlation between the generated data contexts, which means longer memory length. The target *y*_*k*_ is given by the following recursive formula:

4 $$\begin{array}{*{20}{ll}} {u\left( k \right)} \, = \, {rand\left[ {0,0.5} \right],} \\ {U\left( k \right)} \, = \, {2 \ast u\left( k \right),} \\ \quad\,\,{y_k} \, = \, {0.3y_{k - 1} + 0.05y_{k - 1}\left( {\mathop {\sum}\nolimits_i^{n - 1} {y_{k - i - 1}} } \right) + 1.5u_{k - n + 1}u_k + 0.1} \end{array}$$

Different *n* values represent different correlations. Our new model is designed for pattern classification tasks that need strong nonlinear mapping ability; therefore, it sacrifices a certain memory capacity in this special RC framework. We choose *n* = 1, which indicates that the current input is only associated with the last previous response. For the regularization, training, and testing of the dynamic system modeling task, we used two samples with a length of 300 points as the dataset, one for the training and one for the testing. To calculate the memory capacity of the RC system, we calculated our output signal error using the NMSE, which is defined as follows:

5 $${\rm{NMSE}} = \frac{{\mathop {\sum}\nolimits_{k = 1}^m {\mathop {\sum}\nolimits_{i \in {{{\mathrm{O}}}}} {\left( {p_i\left( k \right) - y_i\left( k \right)} \right)^2} } }}{{\mathop {\sum}\nolimits_{k = 1}^m {\mathop {\sum}\nolimits_{i \in {{{\mathrm{O}}}}} {y_i^2\left( k \right)} } }}$$

where *p*(*k*) is the predicted signal, *y*(*k*) is the original signal, and *m* is the number of time steps in the target function.

### Readout function training via ridge regression

The reservoir readout layer was constructed by a linear regression algorithm. We chose ridge regression with Tikhonov regularization to prevent data from overflowing during training and adjusted the weights to minimize the mean squared error between *y* and ***y***_***t***_.

6 $$\begin{array}{*{20}{ll}} {y\left( t \right)} \, = \, {{{{\boldsymbol{w}}}}^{{{\boldsymbol{T}}}}{{{\boldsymbol{x}}}}\left( t \right),} \\ \quad{{{\boldsymbol{w}}}} \, = \, {{{{\boldsymbol{y}}}}_{{{\boldsymbol{t}}}}X^T(XX^T + \lambda I)^{ - 1}} \end{array},$$

where ***w*** is a vector of weights, ***y***_**t**_ is the target vector, *X* is the data matrix that contains *y*(*t*) and ***x***(***t***), and *λ* is the regularization coefficient.
